# Organization and characterization of genetic regions in *Bacillus subtilis* subsp. *krictiensis* ATCC55079 associated with the biosynthesis of iturin and surfactin compounds

**DOI:** 10.1371/journal.pone.0188179

**Published:** 2017-12-21

**Authors:** Young Tae Kim, Byung Keun Park, Sung Eun Kim, Won Jung Lee, Jae Sun Moon, Min Seop Cho, Ho-Yong Park, Ingyu Hwang, Sung Uk Kim

**Affiliations:** 1 Division of Systems Biology and Bioengineering, Korea Research Institute of Bioscience and Biotechnology, Daejeon, Republic of Korea; 2 Green Biotech Co., Paju, Republic of Korea; 3 Department of Agricultural Biotechnology, Seoul National University, Seoul, Republic of Korea; Dong-A University, REPUBLIC OF KOREA

## Abstract

*Bacillus subtilis* subsp. *krictiensis* ATCC55079 produces the cyclic lipopeptide antibiotics iturin A–F as well as several surfactins. Here, we analyzed and characterized the biosynthetic genes associated with iturin and surfactin production in this strain. We aligned the sequences of each iturin and surfactin synthetase ORF obtained from a genomic library screen and next generation sequencing. The resulting 37,249-bp and 37,645-bp sequences associated with iturin and surfactin production, respectively, contained several ORFs that are predicted to encode proteins involved in iturin and surfactin biosynthesis. These ORFs showed higher sequence homologies with the respective iturin and surfactin synthetase genes of *B*. *methylotrophicus* CAU B946 than with those of *B*. *subtilis* RB14 and *B*. *subtilis* ATCC6633. Moreover, comparative analysis of the secondary metabolites produced by the wild-type and surfactin-less mutant (with a spectinomycin resistance cassette inserted into the *srfAB* gene within the putative surfactin gene region) strains demonstrated that the mutant strain showed significantly higher antifungal activity against *Fusarium oxysporum* than the wild-type strain. In addition, the wild-type strain-specific surfactin high performance liquid chromatography (HPLC) peaks were not observed in the surfactin-less mutant strain. In contrast, the iturin A peak detected by HPLC and liquid chromatography-mass spectrometry (LC/MS) in the surfactin-less mutant strain was 30% greater than that in the wild-type strain. These results suggested that the gene cluster we identified is involved in surfactin biosynthesis, and the biosynthetic pathways for iturin and surfactin in *Bacillus* strains producing both iturin and surfactin may utilize a common pathway.

## Introduction

The increasing prevalence of fungicide-resistant fungal strains and public concern over the harmful environmental effects of agrochemicals have prompt the concept of environmentally friendly biological control agents as alternatives to or complements of agrochemicals [1–3]. Various bacterial strains have been used as biological control agents to suppress plant diseases [4–7] and postharvest decay of fruits and vegetables [8, 9]. *Bacillus* species, which produce antibiotics that inhibit plant pathogens [6–8] and are environmentally safe, are among the most remarkable bacterial control agents [8]. In fact, *Bacillus*-based products constitute about half of the commercially available bacterial control agents [10].

*Bacillus subtilis* is a gram-positive bacterium that produces various nonribosomally synthesized cyclic lipopeptides. These compounds share a cyclic structure consisting of a β-amino or β-hydroxy fatty acid integrated into a peptide moiety [11]. The prominent differences among cyclic lipopeptides are the type and sequence of the amino acids in the peptide and the branching of the fatty acid chain. Cyclic lipopeptides are classified into three families, the iturin [10, 12], fengycin and plipastatin [13], and surfactin families [14]. Members of the iturin family, such as iturin, bacillomycin, and mycosubtilin, show potent antifungal activity and are heptapeptides linked to a β-amino fatty acid chain [15–17]. Members of the fengycin and plipastatin family are decapeptides with a β-hydroxy fatty acid, while members of the surfactin family are heptapeptides with a β-hydroxy fatty acid [6]. Several studies on iturin and surfactin [18], iturin and fengycin [6], and surfactin and fengycin [19] have shown that these lipopeptides have synergistic functions. Moreover, some *Bacillus* strains have been shown to simultaneously produce all three lipopeptide families [6, 19–22].

The biosynthesis genes that encode the proteins that produce various lipopeptides of the iturin family have been cloned and sequenced, including the mycosubtilin synthetase of *B*. *subtilis* ATCC6633 [15], the iturin A operon of *B*. *subtilis* RB14 [17], and the bacillomycin D operons of *B*. *amyloliquefaciens* FZB42 [21] and *B*. *subtilis* AU195 [16]. In addition, the gene clusters encoding proteins associated with iturin A and surfactin synthesis have been widely investigated [17, 23–26]. The iturin operon was reported to be more than 38 kb long and composed of four open reading frames, *ituD*, *ituA*, *ituB*, and *ituC* [17], while the surfactin gene cluster consisted of four open reading frames [26]. However, studies of the iturin and surfactin biosynthesis genes in *Bacillus* strains producing both iturin and surfactin are extremely limited, and their sequences differ significantly among strains [17, 25]. Therefore, further studies of iturin and surfactin biosynthesis, including the sequencing of complete iturin and surfactin biosynthesis genes are needed.

In a previous study, we reported that *B*. *subtilis* subsp. *krictiensis* ATCC55079 produces six kinds of iturins [27], has suppressive effects against various phytopathogenic fungi, and shows potential for use as a biological control agent [28, 29]. To compare the iturin and surfactin biosynthesis genes in this strain with the corresponding genes of other *Bacillus* strains, we analyzed the gene clusters associated with iturin and surfactin biosynthesis using a genomic library and next generation sequencing (NGS). To determine whether the identified genes are essential for iturin or surfactin biosynthesis, we generated a mutant strain with a spectinomycin resistant gene cassette inserted into the genes of the wild-type *Bacillus* strain by homologous recombination. Then, the secondary metabolites produced by the wild-type and *srfAB* mutant *B*. *subtilis* subsp. *krictiensis* ATCC55079 strains were analyzed by HPLC and LC-MS.

## Materials and methods

### Bacterial strains, plasmids, and culture conditions

*Bacillus subtilis* subsp. *krictiensis* ATCC55079, the iturin and surfactin-producing strain used in this study, was isolated from soil [27–29]. *B*. *subtilis* 168 [26] and *Escherichia coli* HB101 were obtained from the Korea Research Institute of Bioscience and Biotechnology (Daejeon, Korea). *E*. *coli* DH5α, plasmid pTZ18R (Amersham Pharmacia Biotech), and cosmid pLAFR3 were obtained from Seoul National University (Seoul, Korea) and were used for routine cloning and sequencing. Cosmid pLAFR3 and *E*. *coli* HB101 were utilized to construct a genomic library. The pBC KS(+) (Stratagene) vector and the mini-Tn10 delivery vector pIC333 were used for homologous recombination. All bacteria were stored at -70°C in 20% (vol/vol) glycerol. *Fusarium oxysporum*, *Magnaporthe grisea*, and *Trichophyton mentagrophytes*, which were used for the antifungal activity bioassays, were maintained at 25°C on potato dextrose agar and Sabouraud dextrose agar. The details for all the strains, cosmids, and plasmids used in this study are listed in Table 1.

**Table 1:** Strains and plasmids used in this study.

Strain or plasmid	Description	Source or reference
Bacterial strains		
*Escherichia coli* DH5α	F^-^ Φ80d*lacZ*ΔM15 *rec*A1 *end*A1 *gyr*A96 *thi-*1 *hsd*R17 (r_k_^-^ m_k_^+^) *sup*E44 *rel*A1*deo*RΔ(*lac*ZYA-*arg*F) U169	Promega, Madison, WI
*Escherichia coli* HB101	F^-^ *hsd*S20 (r_B_^-^ m_B_^-^) *rec*A13 *ara-*14 *pro*A2 *lac*Y1 *galK2rps*L20 (Str^r^) *xyl-*5 *mtl-*1 *sup*E44 *thi*-1 *leu*B6	Promega, Madison, WI
*Bacillus subtilis* 168	*trpC2*	[26]
*Bacillus subtilis* subsp. *krictiensis* ATCC55079	Wild-type	[28]
Fungi		
*Fusarium oxysporum*	Wild-type	This study
*Magnaporthe grisea*	Wild-type	This study
*Trichophyton mentagrophytes*	Wild-type	This study
Plasmids		
pTZ18R	Amp^r^ plasmid carrying the T7 *g10* promoter	[31]
pLAFR1	pRK290 containing the *cos* site	[32]
pLAFR3	pLAFR1 containing an *Hae*II fragment of pUC8	[32]
pIC333	Mini-Tn10 delivery vector	[33]
pJJ5, pJJ71, pJJ121, pJJ815	Genomic library clones constructed using cosmid vector pLAFR3	This study
pJJ121E2	16.4-kb *Eco*RI-digested pJJ121 fragment inserted into pTZ18R	This study
pJJ121E3	4.8-kb *Eco*RI-digested pJJ121 fragment inserted into pTZ18R	This study
pJJ815E4	3.7-kb *Eco*RI-digested pJJ815 fragment	This study
pJJ121E2-2	7.9-kb *Eco*RI and *Sal*I-digested pJJ121E2 fragment inserted into pBC KS(+)	This study
pJJ121E2-1	*Bam*HI and *Xba*I-digested pIC333 fragment inserted into the *Cla*I site of pJJ121E2-2	This study

All *B*. *subtilis* strains and *E*. *coli* DH5α were incubated at 30°C overnight in Luria-Bertani (LB) broth without or with spectinomycin (100 μg/mL; Sigma). The medium used to culture the *Bacillus* cyclic lipopeptide-producing strains was a complex medium containing sucrose [30.0 g/L], soytone [10.0 g/L], yeast extract [5.0 g/L], K_2_HPO_4_ [0.5 g/L], MgSO_4_ [2.0 g/L], MnCl_2_ [4.0 mg/L], CaCl_2_ [5.0 mg/L], and FeSO_4_·7H_2_O [25.0 mg/L] in distilled water, and adjusted to pH 7.0. For transformation, Spizizen’s minimal medium [30] containing 50% glucose [10 ml/L], 2% casein hydrolysate [10.0 ml/L], 10% yeast extract [10.0 ml/L], 1 M MgCl_2_ [6.0 g/L], KH_2_PO_4_ [6.0 g/L], K_2_HPO_4_ [14.0 g/L], (NH_4_)SO_4_ [2.0 g/L], Na_3_ citrate·2H_2_O [1.0 g/L], and MgSO_4_ [0.2 g/L] was used.

### Genomic DNA library construction

Genomic DNA was isolated from *Bacillus subtilis* subsp. *krictiensis* ATCC55079 according to a previously described method [30]. The genomic library was constructed using the cosmid vector pLAFR3 and Gigapack III XL packaging extract (Stratagene). Genomic DNA fragments greater than 20 kb in length, which were obtained by partial digestion with *Sau3*AI, were ligated to pLAFR3 that was digested with *Bam*HI and dephosphorylated. The ligation mixture was packaged with Gigapack III XL packaging extract and transfected into *E*. *coli* HB101 cells according to the manufacturer’s protocol.

### Cloning putative surfactin biosynthesis genes from the genomic library

To obtain the surfactin biosynthesis genes from the wild-type *B*. *subtilis* genomic library, two PCR primers were designed using the sequence of the surfactin biosynthesis genes of *B*. *subtilis* 168, which was used for the *Bacillus* genome project and contains a surfactin synthetase gene. The primers were synthesized and purified by Bioneer and the PCR was performed on an i-Cycler (Bio-Rad). To clone the putative surfactin biosynthesis genes from the genomic DNA of *B*. *subtilis* 168 and *B*. *subtilis* subsp. *krictiensis*, we amplified the genes by PCR using primers B9 (5′- GCAAAATTTCCGGACAGCGGGATAT-3ʹ) and B10 (5′-TCGATCCGGCCGATGTATTCGAT-3ʹ). Approximately 100 ng of genomic DNA was added to a 50 μL reaction mixture containing 10 mM Tris-HCl (pH 9.0), 40 mM KCl, 1.5 mM MgCl_2_, 10pmol each of primer, 250 μM dNTPs, and 1 unit of Taq polymerase (AccuPower PCR PreMix, Bioneer). The reactions were performed with the following cycling conditions: an initial denaturation step for 5 min at 95°C, 30 cycles of denaturation for 1 min at 95°C, annealing for 1 min at 42°C, and extension for 2 min at 72°C, with a final extension for 5 min at 72°C. Then, an aliquot (20 μL) of the amplification products was separated on a 1% agarose gel (SeaKem LE agarose, Lonza) in 1× TAE buffer (40 mM Tris-acetate, 1 mM EDTA, pH 8.0). A DNA fragment of approximately 1.8 kb, which is the same size as the products obtained from *B*. *subtilis* 168, was detected in the wild-type *B*. *subtilis* subsp. *krictiensis* strain. The sequence of this product was determined and compared to that of other cyclic lipopeptide synthetase genes. Then, this 1.8-kb gene product was used as a probe in a colony hybridization experiment to select clones containing the putative surfactin biosynthesis genes from a genomic library of the wild-type *B*. *subtilis* strain. For the hybridization experiment, the 1.8-kb PCR amplification product from *B*. *subtilis* subsp. *krictiensis* was purified using the QIAquick gel extraction kit (Qiagen) and then labeled with [α-^32^P]-dCTP using the Prime-a-gene labeling system (Promega). Colony hybridization and Southern hybridization with the labeled probe were performed as described previously [34].

### Sequencing of the cosmid clone containing the putative surfactin biosynthesis genes

Positive cosmid clones, pJJ815 and pJJ121, were selected by colony hybridization and Southern hybridization. Restriction enzyme maps of the cosmid clones were constructed by digestion with *Eco*RI and *Sma*I, and then *Eco*RI digested fragments of the cosmid clones were subcloned into the high-copy vector pTZ18R. The sequences of both strands of the vector constructs were confirmed by sequencing on an ABI 3730XL capillary DNA sequencer (Solgent), and the nucleotide sequencing results were analyzed by using NCBI BLAST and CLUSTALW.

### Disruption of the region containing putative surfactin biosynthesis genes by homologous recombination

A mutant in which the putative surfactin biosynthesis gene was disrupted (a surfactin-less mutant) was constructed by double crossover homologous recombination. To generate this mutant, a 7.9-kb *Eco*RI and *Sal*I-digested pJJ121E2 fragment containing the putative surfactin biosynthesis genes was cloned into the pBC KS(+) vector to construct pJJ121E2-2. Then, a *Bam*HI and *Xba*I fragment containing a spectinomycin resistance gene cassette was excised from pIC333 and *Cla* I sites were introduced by PCR. Then, the *Bam*HI-*Xba*I fragment containing the *Cla*I sites was ligated into the *Cla*I site of the putative surfactin biosynthesis gene in pJJ121E2-2, to create pJJ121E2-1, which contains a surfactin biosynthesis gene disrupted by a spectinomycin resistance gene cassette. The pJJ121E2-1 plasmid was transformed into wild-type *B*. *subtilis* subsp. *krictiensis* grown in Spizizen’s minimal medium as follows. Wild-type *B*. *subtilis* subsp. *krictiensis* was inoculated into 2 mL of Spizizen’s medium and cultivated at 30°C with shaking at 200 rpm for 16 h. An aliquot of this culture was inoculated into fresh Spizizen’s medium and grown at 30°C with shaking at 200 rpm for 16 h until the cultures reached an absorbance at 580 nm (A_580_) of 1.0. Then, the wild-type strain was transformed with pJJ121E2-1 plasmid, which contains a mini-Tn10 transposon, to replace the internal 7.9-kb region of the *srfAB* fragment in the genome with the fragment in pJJ121E2 containing a spectinomycin gene cassette via homologous recombination by selecting spectinomycin-resistant transformants. For the transformation, 1 μg of pJJ121E2-1 was added to 0.5 mL of culture. After incubation at 30°C with shaking at 200 rpm for 60 min, the mixture was spread on an LB agar plate containing 100 μg/mL spectinomycin and incubated at 30°C for 24 h. Transformants that grew on LB agar containing 100 μg/mL spectinomycin were selected and subjected to Southern blot analysis to verify integration of the vector in the chromosome. Genomic DNAs from several transformants were digested with *Cla*I, separated on a 0.7% agarose gel, and blotted to a nylon membrane (Amersham Pharmacia Biotech). Southern hybridization was performed with a spectinomycin-resistance gene probe labeled with DIG-11-dUTP [34] for 16 h at 65°C. DNA labeling and detection were performed using a DIG DNA labeling and detection kit (Roche, Germany) according to the manufacturer’s instructions.

### Comparison of the antifungal activities of the wild-type and surfactin-less mutant *B*. *subtilis* subsp. *krictiensis* strains

The antifungal activities in the culture broth from the wild-type and surfactin-less mutant strains were determined by the agar diffusion method [35]. Mycelial or spore suspensions of test fungi were mixed with a soft agar overlay of 0.8% potato dextrose or Sabouraud dextrose agar and added to potato dextrose agar and Sabouraud dextrose agar plates, respectively. After solidification of the agar overlay, the plates were used in the bioassay. Sterile, stainless steel cylinders (8 mm outer diameter × 10 mm long, Fisher) were placed on the surface of the agar plates, and test samples of the culture broth from the wild-type and mutant strains were loaded into the sterile cylinders, and the plates were incubated at 25°C for 2 days. Then, the diameter of the inhibitory zone on the plates was measured and recorded in millimeters. In addition, commercially available iturin A and surfactin were used on plates containing the test fungi as positive controls.

### Comparative analysis of the secondary metabolites produced by the wild-type and surfactin-less mutant *B*. *subtilis* strains

To assess iturin and surfactin production, the *Bacillus subtilis* strains were grown in the complex medium described above at 30°C for 3 days. The cells were removed by centrifugation at 8,000 × g for 10 min, and the supernatants were adjusted to pH 3 and incubated overnight at 4°C to precipitate the lipopeptides. The precipitates were centrifuged, dissolved in 1M Tris-HCl buffer (pH 7.4), and extracted three times with butanol. The butanol layers were evaporated *in vacuo*, dissolved in methanol, and then filtered through a 0.45-μm filter. The secondary metabolites obtained from the culture broth of the wild-type *B*. *subtilis* subsp. *krictiensis* and surfactin-less mutant strains were analyzed by high performance liquid chromatography (HPLC; Agilent 1100) with a C_18_ column (YMC-pack Pro, 4.6 × 250 mm, 5 μm; YMC). The peaks at 210 nm were detected with a UV detector. The column was eluted with a gradient of CH_3_CN (A)/0.05% trifluoroacetic acid in water (B) at a flow rate of 1 mL/min as follows: 20–60% A/80-40% B (v/v) for 50 min, 60–80% A/40-20% B (v/v) for 5 min, 80–100% A/20-0% B (v/v) for 30 min, 100% A/0% B (v/v) for 3 min, and 20% A/80% B (v/v) for 2 min. Authentic iturin and surfactin (Sigma) were used as references.

To determine the molecular weights of the iturin and surfactin peaks from wild-type *B*. *subtilis* subsp. *krictiensis* that were detected by HPLC, butanol extract-evaporated culture broth was analyzed by using a Nanospace SI-2 HPLC (Shiseido, Tokyo, Japan) and an LCQ Deca XP ion trap mass spectrometer (Thermo Finnigan, San Jose, CA) equipped with an electrospray ionization interface at the Korea Basic Science Institute (Seoul). The column used was a Phenomenex C_18_ column (1.0 × 150 mm, 5 μm; Phenomenex, U. S. A.), which was eluted with CH_3_CN containing 0.1% formic acid (A) and water containing 0.1% formic acid (B) at a flow rate of 50 μL/min as follows: 35% A/65% B (v/v) for 5 min, 35–100% A /65-0% B (v/v) for 75 min, 100% A /0% B (v/v) for 5 min, 100–35% A /0-65% B (v/v) for 5 min, and 35% A/65% B (v/v) for 10 min. Mass spectra were obtained in positive ion mode with m/z values ranging from 50 to 2,000.

### Next-generation sequencing of iturin biosynthesis genes

Whole genome sequence of wild-type *B*. *subtilis* subsp. *krictiensis* was obtained to identify the iturin biosynthesis genes by sequencing on an Illumina Hiseq 2500 sequencer (Teragen Etex Bio Institute, Korea), and the nucleotide sequencing results were analyzed with A5 software (ver. 2015522).

### Nucleotide sequence accession numbers

The nucleotide sequences of the iturin and surfactin biosynthesis genes from *B*. *subtilis* subsp. *krictiensi*s have been deposited in GenBank (accession numbers KU170613 and KC454625, respectively).

## Results

### Cloning and organization of cosmid clones containing putative surfactin biosynthesis genes from a genomic library of wild-type *B*. *subtilis*

**Fig 1:**
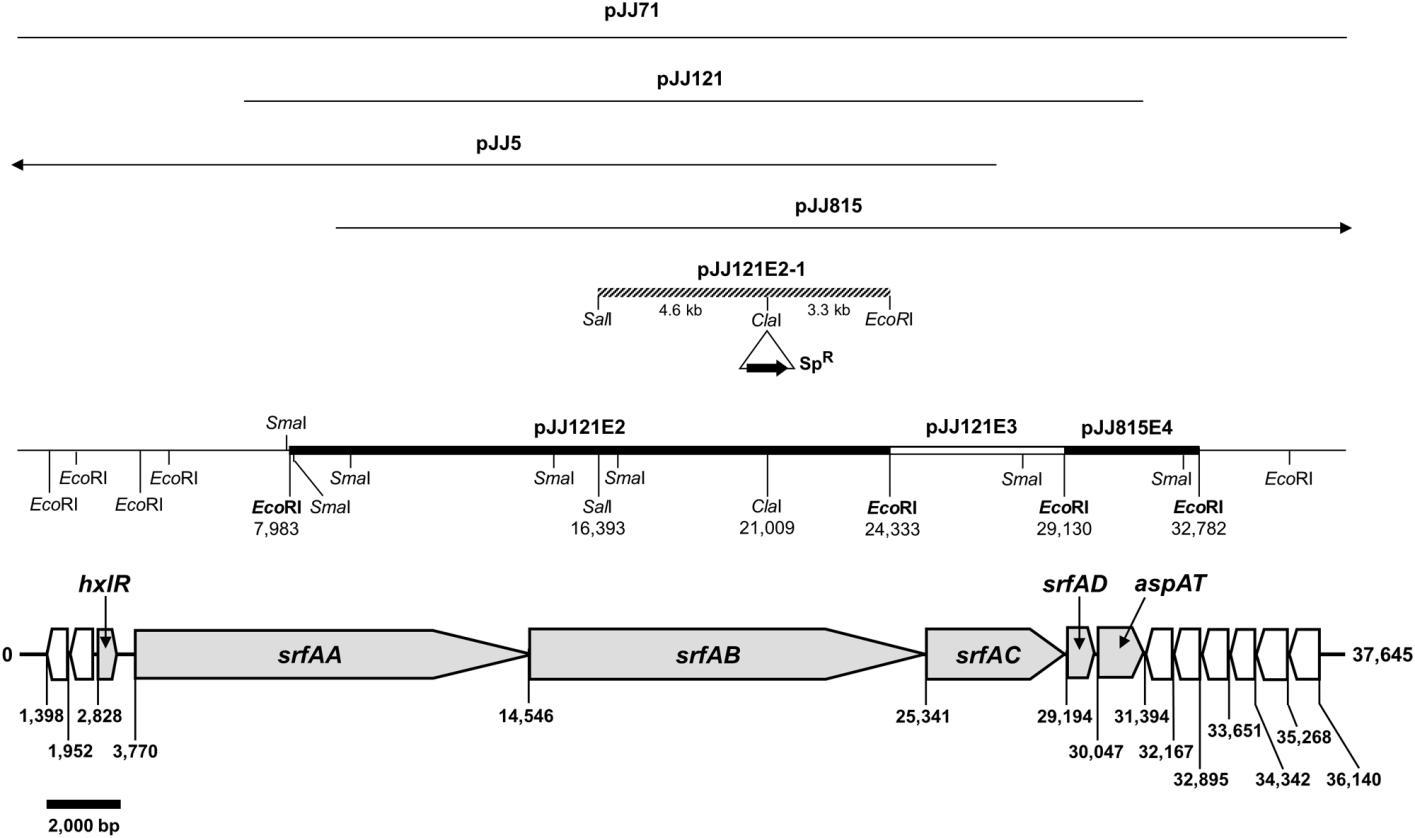
Organization of the ORFs and restriction map of the putative surfactin biosynthesis genes cloned from *Bacillus subtilis* subsp. *krictiensis* ATCC55079. The surfactin genes are designated as *srfAA*, *srfAB*, *srfAC*, and *srfAD*. The spectinomycin resistance gene from the mini-Tn10 in pIC333 is designated as Sp^R^.

To clone the biosynthesis genes responsible for surfactin production, a screening was performed of a genomic library from wild-type *B*. *subtilis* subsp. *krictiensis* ATCC55079. The structures, molecular weights, and lengths of the biosynthetic genes of iturin and surfactin are very similar. In addition, some *Bacillus* strains produce both iturin and surfactin simultaneously [17, 36]. Based on these observations, surfactin and iturin biosynthesis are thought to share a common pathway up to the established steps, after which, these two cyclic lipopeptides are synthesized via separate pathways. Therefore, we attempted to clone the surfactin biosynthesis genes of *B*. *subtilis* subsp. *krictiensis* using the sequences of the surfactin biosynthesis genes from *B*. *subtilis* 168 [26], which was used in the *Bacillus* genome sequencing project. The1.8-kb products amplified from wild-type *B*. *subtilis* subsp. *krictiensis* and *B*. *subtilis* 168 genomic DNA showed 66–99% homology to the sequences of several cyclic lipopeptide synthetase genes, such as surfactin synthetase, peptide synthetase, and lichenysin synthetase. Several positive cosmid clones with 30-kb–40-kb inserts were identified via colony hybridization with a radiolabeled 1.8-kb PCR product as a probe, and four clones (pJJ5, pJJ71, pJJ121, and pJJ815) were finally selected and used to construct a restriction enzyme map of the cosmid clones (Fig 1).

### Sequencing of cosmid clones containing putative surfactin biosynthesis genes

**Fig 2:**
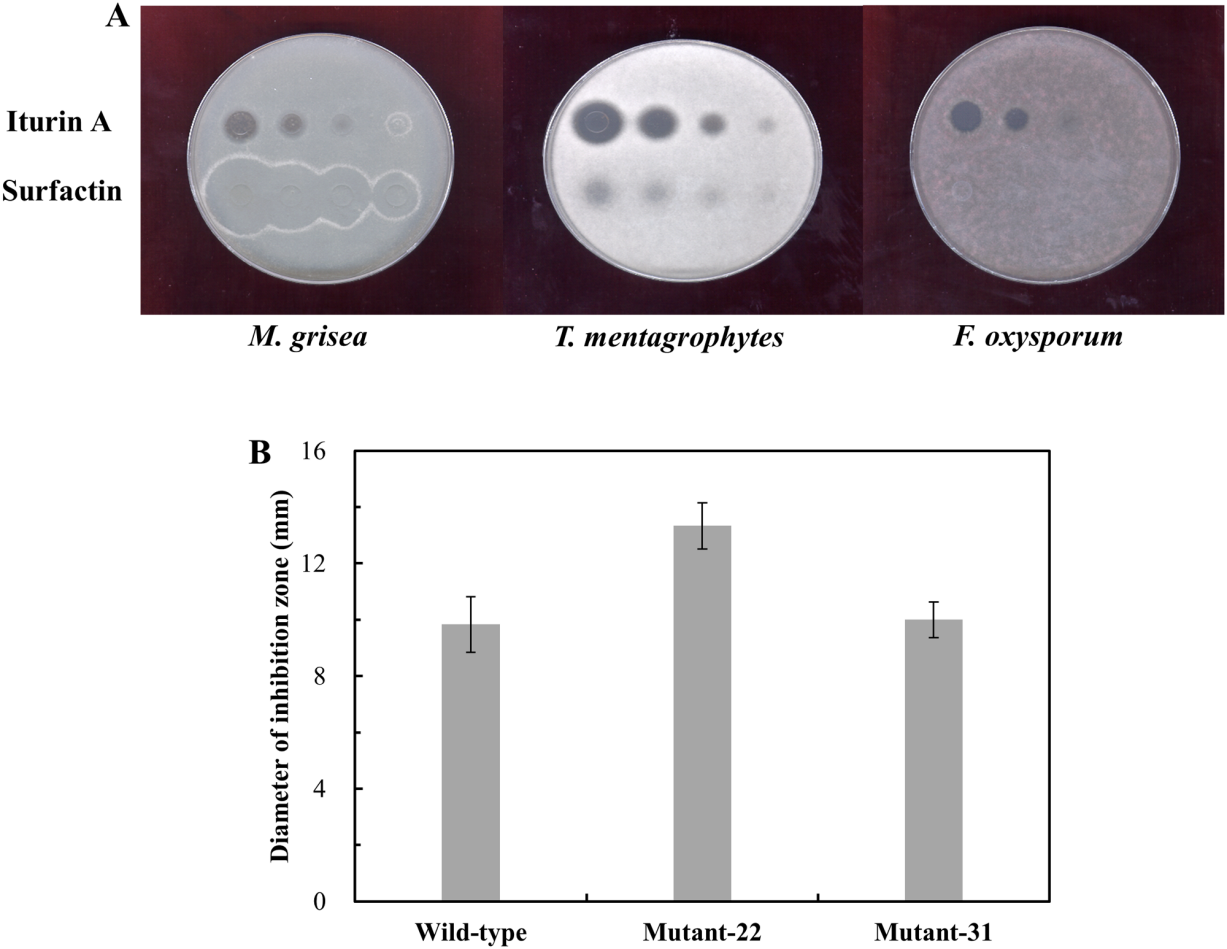
Comparison of the antifungal activities of the wild-type and surfactin-less mutants *B*. *subtilis* subsp. *krictiensis* ATCC55079 strains against *F*. *oxysporum*. (A) Comparison of the antifungal activities of authentic iturin (top) and surfactin (bottom) against various fungi. Two-fold serially dilutions of iturin and surfactin were loaded into PDA plates containing *M*. *grisea* (concentration range, 1.56–12.5 μg/ml; left), *F*. *oxysporum* (6.25–50 μg/mL; right), and a Sabouraud plate containing *T*. *mentagrophytes* (3.12–25 μg/mL; center). (B) Comparison of the antifungal activities of the wild-type, mutant-22, and mutant-31strains. Antifungal activities against *F*. *oxysporum* were examined using culture broth (250 μL) from each strain. Data are expressed as means ± SD for separate experiments performed in sextuplicate.

To determine the sequence of the putative surfactin biosynthesis genes of *B*. *subtilis* subsp. *krictiensis*, the selected cosmid clones were subcloned into a pTZ18R vector, and the sequence of the subclones was determined. The sequence of the putative surfactin biosynthesis genes from *B*. *subtilis* subsp. *krictiensis* was 37,645 bp in length and contained 14 open reading frames (ORFs; Fig 1). Six of the ORFs are in the same orientation, whereas the others are in the opposite orientation. Two ORFs, *hxlB* and *hxlA*, located upstream of the putative surfactin region have homology to the *B*. *subtilis* 168 genes *hxlB* (72%) and *hxlA* (79%) [26], and the *B*. *methylotrophicus* CAU B946 (formerly *B*. *amyloliquefaciens* subsp. *plantarum* CAU B946) genes *hxlB* (99%) and *hxlA* (99%) [25, 37], respectively. These genes are thought to encode the sugar phosphate isomerase (*hxlB*) involved in capsule formation and a sugar phosphate synthase (*hxlA)*, which are key enzymes in the ribulose monophosphate pathway, in which compounds containing carbon-carbon bonds are synthesized from single carbon units [38]. The third ORF, designated *hxlR*, is separated from *hxlA* by 217 bp and has significant homology to the *hxlAB* genes in the surfactin synthetase operon of *B*. *subtilis* 168 (79%), an HTH-type transcriptional activator, *hxlRI*, of *B*. *methylotrophicus* CAU B946 (99%), and *hxlR* of *B*. *subtilis* TU-B-10 (79%) [39]. The fourth ORF, located 589 bp downstream of *hxlR*, is 10,755 bp and is designated *srfAA*. *SrfAA* showed 74% and 99% homology to the surfactin synthetase genes (*srfAA*) of *B*. *subtilis* 168 [26] and *B*. *methylotrophicus* CAU B946 [25, 37], respectively. The next gene, designated *srfAB*, which was 10,760 bp in size, showed 74% and 99% homology to the surfactin synthetases (*srfAB*) of *B*. *subtilis* 168 and *B*. *methylotrophicus* CAU B946, respectively. *SrfAC*, which is located 35 bp downstream of *srfAB*, showed 87% and 99% homology to the surfactin synthetases (*srfAC*) of *B*. *subtilis* 168 and *B*. *methylotrophicus* CAU B946. The seventh ORF, designated *srfAD*, exhibited 75% and 99% homology to the surfactin synthetase thioesterases (*srfAD*) of *B*. *subtilis* 168 and *B*. *methylotrophicus* CAU B946, respectively. The next gene, *aspAT*, shared more than 98% homology with the aspartate aminotransferases *aspB3* [24] and *aspAT* of *B*. *subtilis* and *B*. *methylotrophicus* CAU B946, respectively. The *sfp* gene, located in 37 bp downstream of *aspAT*, encodes a 4′-phosphopantetheinyl transferase and showed 72% and 73% homology to the *sfp* of *B*. *subtilis* TU-B-10 and *B*. *subtilis* 168, respectively, and 99% homology to *B*. *methylotrophicus* CAU B946. The next gene, *yczE*, which is predicted to encode an inner membrane protein regulating antibiotic production, showed 74% homology to *yczE* of *B*. *subtilis* 168 and >98% homology to *yczE* of both *B*. *subtilis* [24] and *B*. *methylotrophicus* CAU B946. The next three genes, *yckI*, *yckJ*, and *yckK*, which encode amino acid ABC transporter proteins, showed 81–87% homology to the *tcy* genes of *B*. *subtilis* 168 and 99–100% homology to the *yck* genes of *B*. *methylotrophicus* CAU B946. The final ORF exhibited 75% homology to *bsdA* and *yclA* of *B*. *subtilis* 168 and *B*. *subtilis* TU-B-10 [39], respectively, and 99% homology to *yclA* of *B*. *methylotrophicus* CAU B946. Interestingly, the sequences of *srfAA*, *srfAB*, *srfAC*, and *srfAD* in wild-type *B*. *subtilis* subsp. *krictiensis* showed relatively low homology (74–87%) to the surfactin synthetase operon of *B*. *subtilis* 168 and very high homology (99%) to the surfactin synthetase operon of *B*. *methylotrophicus* CAU B946. Although the surfactin biosynthesis genes cloned from wild-type *B*. *subtilis* subsp. *krictiensis* are highly homologous to the surfactin synthetase in *B*. *methylotrophicus* CAU B946 [25, 37], *B*. *subtilis* subsp. *krictiensis* has already been shown to produce various iturin compounds by NMR and MS analyses and amino acid composition determination [27, 28] as well as several surfactins. Moreover, the *in vitro* antifungal activities of iturin and surfactin against *F*. *oxysporum* clearly differed (Fig 2A). Based on the differences in antifungal activity and the high levels of sequence homology between wild-type *B*. *subtilis* subsp. *krictiensis* and *B*. *methylotrophicus* CAU B946, we decided to further investigate the putative surfactin biosynthesis genes from the wild-type *B*. *subtilis* subsp. *krictiensis* strain to determine whether these genes were directly involved in surfactin biosynthesis.

### Construction of a surfactin-less *B*. *subtilis* subsp. *krictiensis* mutant

To disrupt the *srfAB* gene in *B*. *subtilis* subsp. *krictiensis*, the pJJ121E2-1 plasmid containing the spectinomycin resistance gene was constructed by homologous recombination. The pJJ121E2-1 plasmid was transformed into wild-type *B*. *subtilis* subsp. *krictiensis*, and several transformants that grew on LB agar plates containing spectinomycin (which should be *srfAB* disruption mutants) were selected. The antifungal activities of these transformants against *F*. *oxysporum* were examined. Two colonies were finally selected, which were designated mutant-22 and -31, and were used for further studies. The mutant-22 and -31 showed inhibition zones of average 13.3 ± 0.8 mm and 10.0 ± 0.6 mm against *F*. *oxysporum*, respectively, whereas the wild-type strain showed zones of average 9.8 ± 0.9 mm. Interestingly, mutant-22 showed significantly higher antifungal activities against *F*. *oxysporum* than the wild-type *B*. *subtilis* subsp. *krictiensis* strain (Fig 2B), whereas mutant-31 showed antifungal activity similar to that of the wild-type strain. The antifungal activities of commercially available iturin A and surfactin against *F*. *oxysporum* were examined as positive controls. As shown in Fig 2A, authentic iturin A showed potent, dose-dependent, inhibitory activity against *F*. *oxysporum* in the range of 6.25 to 50 μg/mL, whereas surfactin exhibited no inhibitory activity. This result suggested that the antifungal activity of wild-type *B*. *subtilis* subsp. *krictiensis* was due to iturin, whereas the increase in the antifungal activity of mutant-22 was caused by disruption of the surfactin biosynthesis genes.

To confirm the double-crossover in the chromosome of the mutant-22 and -31 strains, genomic DNAs were probed with a spectinomycin resistance gene fragment in a Southern hybridization. For the mutant-22 strain, a DNA band was detected, migrating at the same size as the spectinomycin resistance gene (1.5 kb), whereas no band was detected for wild-type *B*. *subtilis* subsp. *krictiensis* DNA (S1 Fig), which confirmed that the spectinomycin resistant gene was inserted into the chromosomal DNA of the mutant-22 strain. However, for mutant-31, the DNA band detected with spectinomycin resistance gene probe showed a different migration rate, suggesting that the spectinomycin gene was incorrectly inserted into the chromosomal DNA of this strain.

### Comparative analysis of the secondary metabolites in the wild-type and mutant-22 strains by HPLC and LC/MS

**Fig 3:**
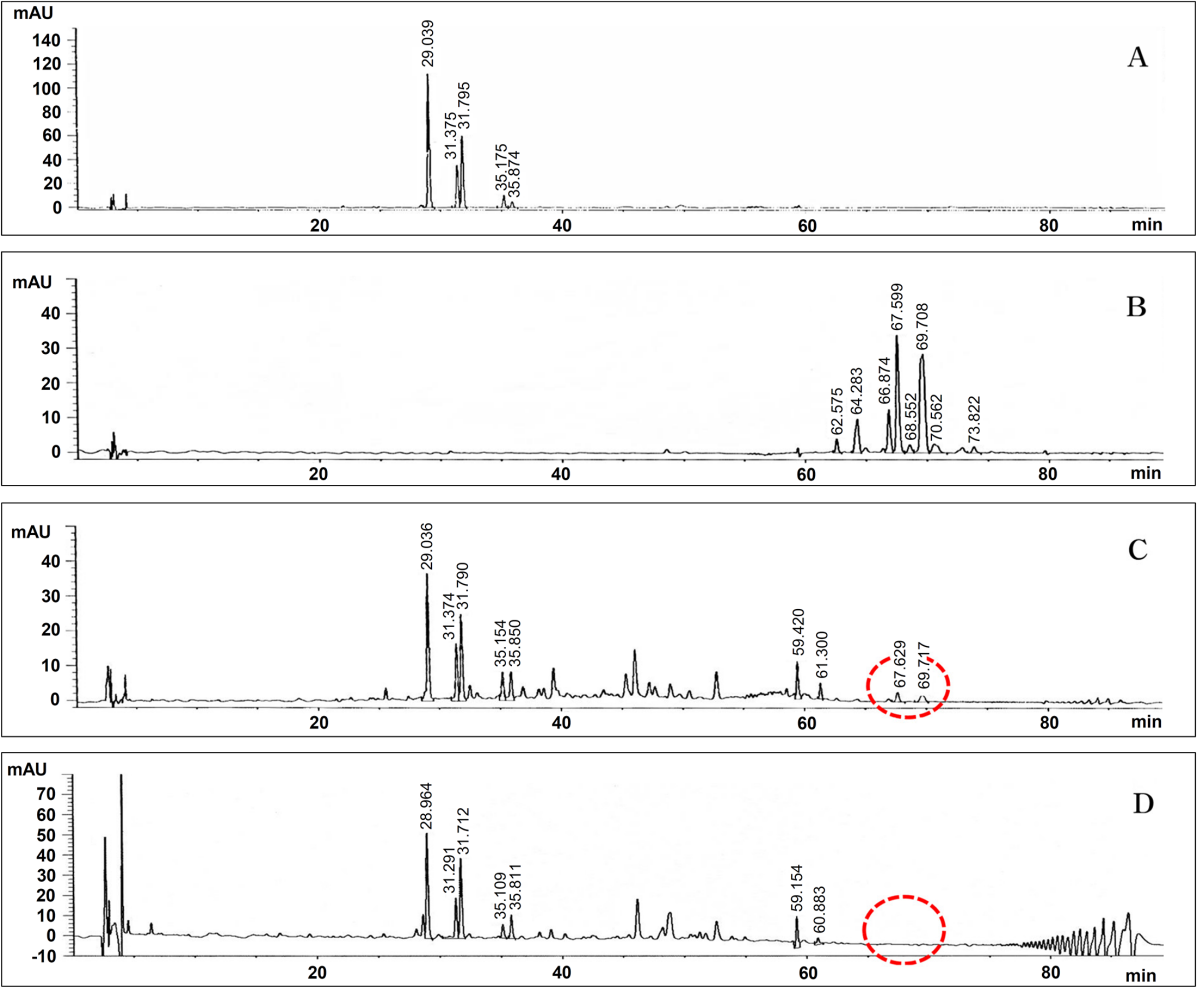
Qualitative HPLC analyses of the iturin and surfactin compounds produced by the wild-type and mutant-22 *B*. *subtilis* subsp. *krictiensis* strains. A: Authentic iturin A (500 μg/mL), B: Authentic surfactin (500 μg/mL), C: Wild-type *B*. *subtilis* subsp. *krictiensis* ATCC55079, D: Surfactin-less mutant-22 strain.

To examine the differences in the cyclic lipopeptides produced by the wild-type and mutant-22 *B*. *subtilis* subsp. *krictiensis* strains, the secondary metabolites extracted from the culture broth of these two strains were analyzed by HPLC. Six iturin compounds peaks were detected in the wild-type strain, and the patterns of these peaks were very similar to those of commercially available authentic iturin A (Fig 3A and 3C) and the same as those of iturin A–F (molecular weights of 1042, 1056, 1056, 1070, 1070, and 1084, respectively), which were previously isolated and identified by various instrumental analyses in our laboratory [27]. Several iturin peaks with the same retention times as the wild-type strain were also observed in mutant-22. However, the small amounts of two surfactin peaks with retention times of 67 and 69 min detected in the wild-type strain were not detected in the mutant-22 strain (Fig 3D), and iturin production by this strain was slightly higher than that of the wild-type strain when the same amount of butanol extract was used in the HPLC analysis (Fig 3D).

**Fig 4:**
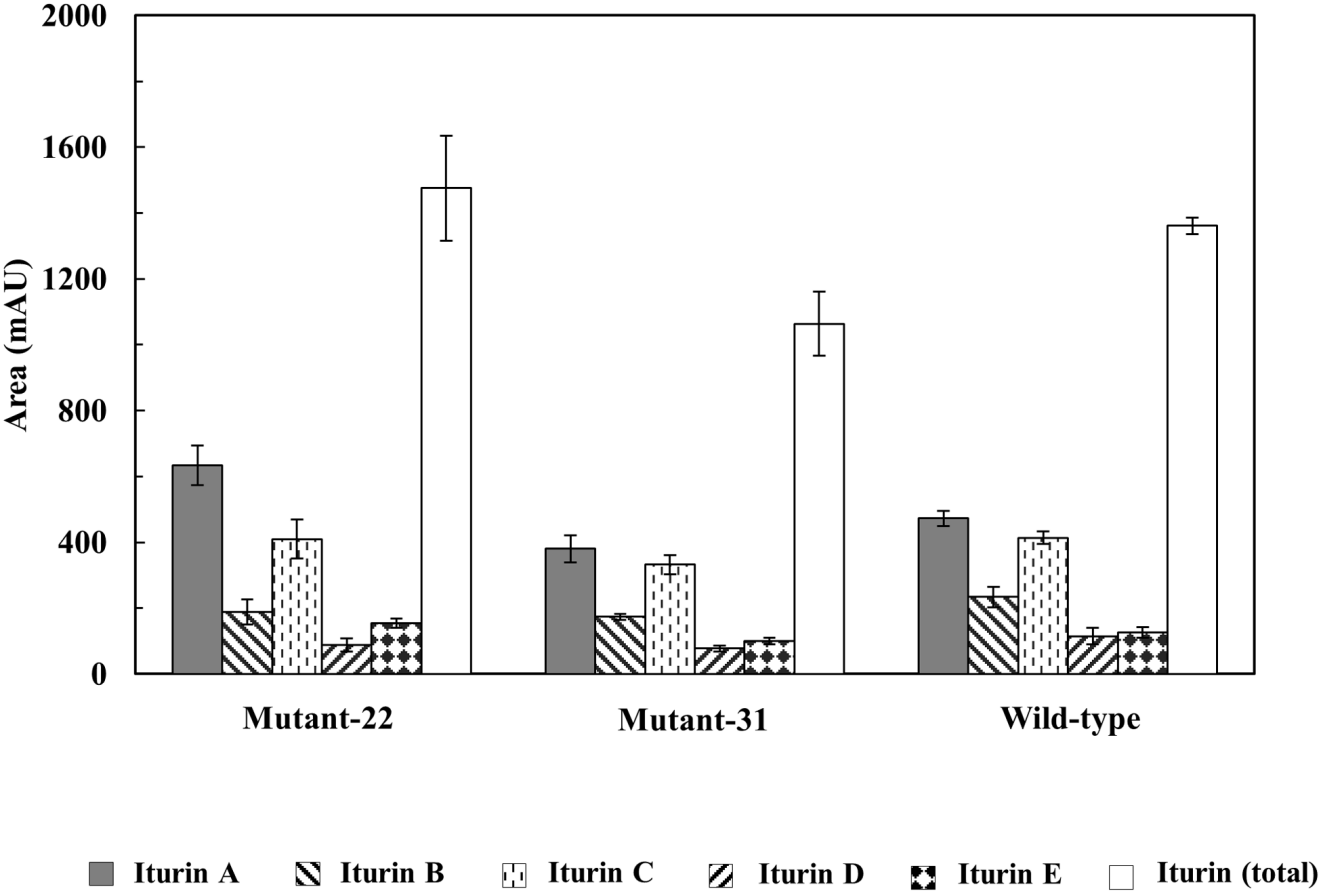
Quantitative analyses of iturin A–E produced by wild-type *B*. *subtilis* subsp. *krictiensis* and the surfactin-less mutants-22 and -31 strains. Data are expressed as means ± SD for separate experiments in quadruplicate.

To analyze the amount of iturin production by the mutant-22 strain, in which a spectinomycin resistance gene was inserted into *srfAB*, the HPLC peak areas of iturin A to E in the wild-type and mutants strains were compared. Iturin F peak areas producing very small amounts in these strains were excluded in the comparative analysis. Interestingly, iturin A production by the mutant-22 strain was markedly higher (by 30 percent), whereas the total iturin production by the mutant-22 strain was just slightly higher than that of the wild-type strain (Fig 4).

In addition, iturin E production was also 20 percent higher, while the iturin B, C, and D production by the mutant strain was lower than that of the wild-type strain. Among these iturins, the iturin A, D, and E show strong antifungal activities [40, 41], whereas iturin B and C have no antifungal activity [41]. This suggests that the increased antifungal activity against *F*. *oxysporum* of mutant-22 (Fig 2B) was due to the increase in iturin A production, considering the small amount of iturin E produced.

To further investigate the iturin and surfactin peaks detected in the wild-type *B*. *subtilis* subsp. *krictiensis* strain, the molecular weights of these peaks were determined by LC-MS. The mass spectra of the six iturin peaks (iturin A–F) detected by HPLC showed quasi-molecular ion peaks [M+H]^+^ at m/z 1,043.5, 1,057.5, 1,057.5, 1,071.5, 1,071.5, and 1,085.5 (Table 2; see S2–S8 Figs), corresponding to molecular weights of 1,042, 1,056, 1,056, 1,070, 1,070, and 1,084, respectively, which was 14 mass units higher than the values of iturin A. The molecular weights of these peaks corresponded to the previously reported molecular masses of iturin A–F [27, 28]. In addition, the mass spectra of various surfactin peaks showed quasi-molecular ion peaks [M+H]^+^ at m/z 1,008.4, 1,022.5, 1,022.5, and 1,036.5), corresponding to molecular weights of 1,007, 1,021, 1,021, and 1,035, respectively (Table 2; see S9–S13 Figs). These results suggested that the identified gene clusters in the genome of *B*. *subtilis* subsp. *krictiensis* were involved in surfactin biosynthesis, even though the mutant-22 strain showed higher antifungal activity against *F*. *oxysporum* than the wild-type strain.

**Table 2:** Cyclic lipopeptide products of the wild-type *B*. *subtilis* subsp. *krictiensis* ATCC55079 strain as detected by LC-MS^a^.

Product and observed mass peaks (*m/z*)		Retention time (min)	Assignment
Iturin	1043.5, 1041.5	22.61	C14- Iturin A [M + H]^+^, [M - H]^-^
	1057.5, 1055.4	24.14	C15- Iturin B [M + H]^+^, [M - H]^-^
	1057.5, 1055.5	26.15	C15- Iturin C [M + H]^+^, [M - H]^-^
	1071.5, 1069.5	26.44	C16- Iturin D [M + H]^+^, [M - H]^-^
	1071.5, 1069.5	27.20	C16- Iturin E [M + H]^+^, [M - H]^-^
	1085.5, 1083.5	28.07	C17- Iturin F [M + H]^+^, [M - H]^-^
Surfactin	1008.5, 1006.6	66.05	C13- surfactin [M + H]^+^, [M - H]^-^
	1022.5, 1020.7	70.88	C14- surfactin [M + H]^+^, [M - H]^-^
	1022.5, 1020.7	71.93	C14- surfactin [M + H]^+^, [M - H]^-^
	1036.5, 1034.5	75.23	C15- surfactin [M + H]^+^, [M - H]^-^

^a^The data were obtained from the supernatant of cells grown in production medium as described in the Materials and Methods. The HPLC peaks presented in S3–S8 and S9 Figs were analyzed by MS spectrometry.

### Sequencing and organization of iturin biosynthesis genes from wild-type *B*. *subtilis*

**Fig 5:**
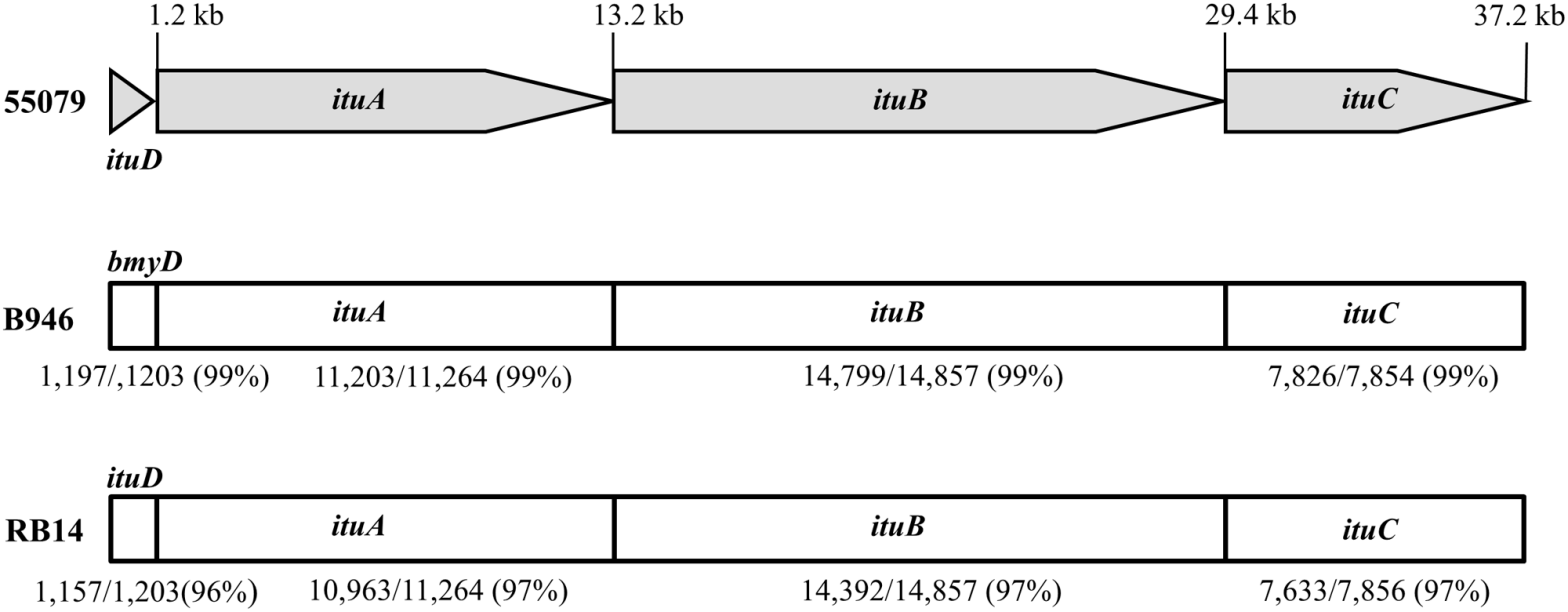
Sequence homologies of the iturin synthetase genes from *B*. *subtilis* subsp. *krictiensis* ATCC55079 with other iturin synthetase genes. The organization and positions of the homologous gene clusters in *B*. *subtilis* RB14 and *B*. *methylotrophicus* CAU B946 were drawn by referring to references 17 and 25.

We sequenced the whole genome of *B*. *subtilis* subsp. *krictiensis* to identify the iturin biosynthesis genes by next generation sequencing. The sequence of the iturin biosynthesis genes in *B*. *subtilis* subsp. *krictiensis* was 37,249 bp in length and contained four iturin biosynthesis genes (Fig 5). *ItuD*, which is 1,203 bp in length, and showed 96% and 99% homology to the malonyl-CoA transacylase gene of *B*. *subtilis* RB14, which contains a complete 38-kb iturin A operon [17], and *B*. *methylotrophicus* CAU B946 [25, 37], respectively. The next gene, designated *ituA*, was 11,951 bp in length and exhibited 97% and 99% homology to the iturin synthetase A of *B*. *subtilis* RB14 and *B*. *methylotrophicus* CAU B946, respectively. The next gene, designated *ituC*, which is located in 89 bp downstream of *ituB* and is 7,853 bp in length, showed 97% and 99% homology to iturin synthetase C genes of *B*. *subtilis* RB14 and *B*. *methylotrophicus* CAU B946 (Fig 5), respectively. The sequences of *ituD*, *ituA*, *ituB*, and *ituC* in *B*. *subtilis* subsp. *Krictiensis* showed relatively high homologies (97–99%) to the iturin synthetase operons of *B*. *subtilis* RB14 and *B*. *methylotrophicus* CAU B946. Although the iturin biosynthesis genes from *B*. *subtilis* subsp. *krictiensis* are more highly homologous to the iturin synthetase in *B*. *methylotrophicus* CAU B946 than to those of *B*. *subtilis* RB14, the slight differences in the sequences of the *ituA* and *ituB* genes, which were 11,952 and 14,858 bp in length, respectively, may reflect a species-specific difference between the two strains.

## Discussion

*B*. *subtilis* subsp. *krictiensis* ATCC55079 produces the potent antifungal cyclic lipopeptides iturin A–F [27, 28]. Recently, we showed, through HPLC and LC/MS analyses, that this strain also produces a small amount of surfactin. Here, we described the identification of the surfactin and iturin biosynthesis genes in this strain, which are located in an approximately 37-kb region. The identified surfactin genes included four ORFs that we designated *srfAA*, *srfAB*, *srfAC*, and *srfAD*. Each ORF showed relatively high homology (74–99%) to the surfactin synthetase genes of *B*. *subtilis* 168 [26] and *B*. *methylotrophicus* CAU B946, which was recently in a genome announcement [25, 37]. To obtain evidence that these putative genes are involved in surfactin biosynthesis, we disrupted *srfAB* in *B*. *subtilis* subsp. *krictiensis* ATCC55079 using a mini-Tn10 transposon-bearing plasmid. The resulting mutant strain (mutant-22) did not produce surfactin, but produced significantly more iturin A than the wild-type strain (Fig 2B). Based on the loss of surfactin production observed in the mutant-22 strain (Fig 3D), we concluded that the putative surfactin biosynthesis genes identified in this study are indispensable for the production of several surfactins.

*SrfAA*, *srfAB*, and *srfAC* encode the condensation and adenylation domains of the large peptide synthetase responsible for the biosynthesis of the peptide chain, as has been observed for other cyclic lipopeptide antibiotics [25, 26], and *srfAD* encodes the thioesterase domain. In addition, other ORFs, including the aspartate aminotransferase gene (*aspAT*), 4′-phosphopantetheine transferase gene (*sfp*), and *yczE*, which encodes the inner membrane protein that regulates antibiotic production, are also located adjacent to *srfAD*. These observations are consistent with a previous report that the 4′-phosphopantetheine transferase (*sfp*) gene, which is defective in the surfactin-producing *B*. *subtilis* 168 strain, is essential for the production of iturin A and surfactin in *B*. *subtilis* RB14 [42, 43] and fengycin in *B*. *amyloliquefaciens* FZB42 [44]. Moreover, they are also in agreement with previous results showing that *sfp* and *yczE* encode essential factors for the production of bacillomycin D, a member of the iturin family, in *B*. *amyloliquefaciens* FZB42 [21, 44]. These results suggest that the production of non-ribosomal cyclic lipopeptide antibiotics in *Bacillus* might be controlled by a common regulatory system [43]. However, the modules in the conserved domains of these surfactin biosynthesis genes were more similar to those in the surfactin operons of *B*. *methylotrophicus* CAU B946 [25, 37] than those of *B*. *subtilis* 168 [26]. In contrast, the iturin biosynthesis genes of *B*. *subtilis* subsp. *krictiensis* are also closely related, but not identical, to those of *B*. *methylotrophicus* CAU B946 and *B*. *subtilis* RB14, which contains a complete 38-kb iturin A operon. Based on these results, the different homologies among the iturin and surfactin biosynthesis genes in *B*. *subtilis* subsp. *krictiensis*, *B*. *subtilis* RB14, *B*. *subtilis* 168, and *B*. *methylotrophicus* CAU B946 might be due to differences in species specificity.

Because the surfactin-less mutant-22 strain, which has a disrupted surfactin biosynthesis gene, exhibited a 30% increase in iturin A production compare to that in the wild-type strain, iturin biosynthesis in *B*. *subtilis* subsp. *krictiensis* might also occur through an alternative pathway other than the one involving genes encoded by the iturin A and surfactin operons previously described [17, 26].

Although we do not have any experimental data on the regulatory mechanism underlying the increased iturin production in the surfactin-less mutant strain, we propose two possible explanations. First, the enhanced iturin production in the mutant strain might be due to increased iturin biosynthesis using the substrate remaining in medium after blocking surfactin biosynthesis. Second, positive or negative regulators of cyclic lipopeptides, such as the ComP/ComA two-component system, DegU, Sfp or YczE, PerR, and Rap proteins and Phr peptides, might be involved in a mechanism that increases iturin production in the surfactin-less mutant strain. ComP/ComA, Sfp, PerR, and Phr are positive regulators of *srfA* transcription [11, 45–48], and overexpression of the *comA* and *sigA* genes was shown to improve iturin production [49]. In addition, DegU and YczE positively regulate the synthesis of bacillomycin D in *Bacillus amyloliquefaciens* strain [50]. Thus, changes in the expression levels of regulators of the iturin operon may enhance iturin production in the surfactin-less mutant. However, at present, identification of the regulators responsible for the increased iturin production in the surfactin-less mutant strain and the detailed mechanism remain to be investigated.

This is the first report of increased antifungal activity in a surfactin-less mutant, which contains a disrupted surfactin biosynthesis gene, which is involved in iturin and surfactin production. However, the ORFs responsible for surfactin biosynthesis in *B*. *subtilis* subsp. *krictiensis* showed low level homology to the surfactin operon of *B*. *subtilis* 168 [26] and high level homology to the surfactin synthetase of *B*. *methylotrophicus* CAU B946 [25, 37]. In addition, *B*. *subtilis* subsp. *krictiensis* exhibited high level homologies to the iturin operons of *B*. *subtilis* RB14 and *B*. *methylotrophicus* CAU B946. We confirmed that the production of these two cyclic lipopeptide antibiotics (iturin and surfactin) may utilize a common pathway up to the previously established steps, which could provide an alternative approach for cloning genes for the production of nonribosomal cyclic lipopeptide antibiotics. Any interaction between the iturin and surfactin biosynthesis genes in *Bacillus* strains producing both iturin and surfactin in the production of cyclic lipopeptides needs to be investigated in future studies.

## Supporting information

S1 Fig Southern hybridization of genomic DNA from wild-type *B*. *subtilis* subsp. *krictiensis* and various transformants using a spectinomycin resistance gene probe. Lanes: 1, genomic DNA from wild-type *B*. *subtilis* subsp. *krictiensis* digested with *ClaI*; 2, 3, 21, 22, 23, and 31, genomic DNAs from various transformants digested with *ClaI*; pJJ121E2-1, the spectinomycin resistance gene from the mini-Tn10 of pIC333 digested with *Xba*I and *Bam*HI.
(DOCX)

S2 Fig HPLC chromatograms of the iturin compounds produced by wild-type *B*. *subtilis* subsp. *krictiensis* ATCC55079. (DOCX)

S3 Fig MS spectrum of iturin A at a retention time of 22.61 min. (DOCX)

S4 Fig MS spectrum of iturin B at a retention time of 24.14 min. (DOCX)

S5 Fig MS spectrum of iturin C at a retention time of 26.15 min. (DOCX)

S6 Fig MS spectrum of iturin D at a retention time of 26.44 min. (DOCX)

S7 Fig MS spectrum of iturin E at a retention time of 27.20 min. (DOCX)

S8 Fig MS spectrum of iturin F at a retention time of 28.07 min. (DOCX)

S9 Fig HPLC spectra and molecular weights of surfactin peaks obtained with authentic surfactin and wild-type *B*. *subtilis* subsp. *krictiensis* ATCC55079. (DOCX)

S10 Fig MS spectrum of C13-surfactin at a retention time of 66.05 min. (DOCX)

S11 Fig MS spectrum of C14-surfactin at a retention time of 70.88 min. (DOCX)

S12 Fig MS spectrum of C14-surfactin at a retention time of 71.93 min. (DOCX)

S13 Fig MS spectrum of C15-surfactin at a retention time of 75.23 min. (DOCX)

## References

[1] Fernández-Ortuño D, Pérez-Garcia A, López-Ruiz F, Romero D, De Vicente A, Torés JA, et al Occurrence and distribution of resistance to QoI fungicides in populations of *Podosphaera fusca* in south central Spain. Eur J Plant Pathol. 2006; 115: 215–222.

[2] McGrath MT. Fungicide resistance in cucurbit powdery mildew: Experience and challenges. Plant Dis. 2001; 85: 236–245. 10.1094/PDIS.2001.85.3.236 30832035

[3] Kiss L. A review of fungal antagonists of powdery mildews and their potential as biological agents. Pest Manage Sci. 2003; 59: 475–483. 10.1002/ps.689 12701710

[4] De Boer M, Bom P, Kindt F, Keurentjes JJB, Van Der Sluis I, Van Loon LC, et al Control of *Fusarium* wilt of radish by combining *Pseudomonas putida* strains that have different disease-suppressive mechanisms. Phytopathology 2003; 93: 626–632. doi: 10.1094/PHYTO.2003.93.5.626 18942986 10.1094/PHYTO.2003.93.5.626

[5] Cazorla FM, Duckett SB, Bergström ET, Noreen S, Odijk R, Lugtenberg BJJ, et al Biocontrol of avocado dematophora root rot by antagonistic *Pseudomonas fluorescens* PCL1606 correlates with the production of 2-hexyl 5-propyl resorcinol. Mol Plant Microbe Interact. 2006; 19: 418–428. doi: 10.1094/MPMI-19-0418 16610745 10.1094/MPMI-19-0418

[6] Romero D, De Vicente A, Rakotoaly RH, Dufour SE, Veening JW, Arrebola E, et al The iturin and fengycin families of lipopeptides are key factors in antagonism of *Bacillus subtilis* toward *Podosphaera fusca*. Mol Plant Microbe Interact. 2007; 20: 430–440. doi: 10.1094/MPMI-20-4-0430 17427813 10.1094/MPMI-20-4-0430

[7] Leclère V, Bécher M, Adam A, Guez JS, Wathelet B, Ongena M, et al Mycosubtilin overproduction by *Bacillus subtilis* BBG100 enhances the organism’s antagonistic and biocontrol activities. Appl Environ Microbiol. 2005; 71: 4577–4584. doi: 10.1128/AEM.71.8.4577-4584.2005 16085851 10.1128/AEM.71.8.4577-4584.2005 PMC1183317

[8] Arrebola E, Jacobs R, Korsten L. Iturin A is the principal inhibitor in the biocontrol activity of *Bacillus amyloliquefaciens* PPCB004 against postharvest fungal pathogens. J Appl Microbiol. 2010; 108: 386–395. doi: 10.1111/j.1365-2672.2009.04438.x 19674188 10.1111/j.1365-2672.2009.04438.x

[9] Sharma RR, Singh D, Singh R. Biological control of postharvest diseases of fruits and vegetables by microbial antagonist: A review. Biol Control. 2009; 50: 205–221.

[10] Ongena M, Jacques P. *Bacillus* lipopeptides: versatile weapons for plant disease biocontrol. Trends Microbiol. 2007; 16: 115–125. 10.1016/j.tim.2007.12.009 18289856

[11] Roongsawang N, Washio K, Morikawa M. Diversity of nonribosomal peptide synthetases involved in the biosynthesis of lipopeptide biosurfactants. Int J Mol Sci. 2011; 12: 141–172. 10.3390/ijms12010141 PMC3039948 21339982

[12] Peypoux F, Guinand M, Michel G, Delcambe L, Das BC, Lederer E. Structure of iturin A, a peptidolipid antibiotic from *Bacillus subtilis*. Biochemistry 1978; 17: 3992–3996. 101232 10.1021/bi00612a018

[13] Umezawa H, Aoyagi T, Nishikiori T, Okuyama A, Yamagishi Y, Hamada M, et al Plipastatins: new inhibitors of phospholipase A2, produced by *Bacillus cereus* BMG202-fF67. I. Taxonomy, production, isolation and preliminary characterization. J Antibiot. 1986; 39: 737–744. 3089997 10.7164/antibiotics.39.737

[14] Peypoux F, Bonmatin JM, Wallach J. Recent trends in the biochemistry of surfactin. Appl Microbiol Biotechnol. 1999; 51: 553–563. 10390813 10.1007/s002530051432

[15] Duitman E H, Hamoen L. W., Rembold M, Venema G, Seitz H, Saenger W, et al The mycosubtilin synthetase of *Bacillus subtilis* ATCC6633: A multifunctional hybrid between a peptide synthetase, an amino transferase, and a fatty acid synthase. Proc Natl Acad Sci. 1999; 96: 13294–13299. 10557314 10.1073/pnas.96.23.13294 PMC23941

[16] Moyne AL, Cleveland TE, Tuzun S. Molecular characterization and analysis of the operon encoding the antifungal lipopeptide bacillomycin D. FEMS Microbiol Lett. 2004; 234: 43–49. doi: 10.1016/j.femsle.2004.03.011 15109718 10.1016/j.femsle.2004.03.011

[17] Tsuge K, Akiyama T, Shoda M. Cloning, sequencing, and characterization of the iturin A operon. J Bacteriol. 2001; 183: 6265–6273. doi: 10.1128/JB.183.21.6265-6273.2001 11591669 10.1128/JB.183.21.6265-6273.2001 PMC100110

[18] Magnet-Dana R, Thimon L, Peypoux F, Ptak M. Surfactin/iturin A interactions may explain the synergistic effect of surfactin on the biological properties of iturin A. Biochimie. 1992; 74: 1047–1051. 1292612 10.1016/0300-9084(92)90002-v

[19] Ongena M, Jourdan E, Adam A, Paquot M, Brans A, Joris B, et al Surfactin and fengycin lipopeptides of *Bacillus subtilis* as elicitors of induced systemic resistance in plants. Environ Microbiol. 2007; 9: 1084–1090. doi: 10.1111/j.1462-2920.2006.01202.x 17359279 10.1111/j.1462-2920.2006.01202.x

[20] Roongsawang N, Thaniyavarn J. Isolation and characterization of a halotolerant *Bacillus subtilis* BBK-1 which produces three kinds of lipopeptides: bacillomycin L, plipastatin, and surfactin. Extremophiles 2002; 6: 499–506. doi: 10.1007/s00792-002-0287-2 12486459 10.1007/s00792-002-0287-2

[21] Koumoutsi A, Chen XH, Henne A, Liesesang H, Hitzeroth G, Franke P, et al Structural and functional characterization of gene clusters directing nonribosomal synthesis of bioactive cyclic lipopeptides in *Bacillus amyloliquefaciens* strain FZB42. J Bacteriol. 2004; 186: 1084–1096. doi: 10.1128/JB.186.4.1084-1096.2004 14762003 10.1128/JB.186.4.1084-1096.2004 PMC344220

[22] Patel H, Tscheka C, Edwards K, Karlsson G, Heerklotz H. All-or-none membrane permeabilization by fengycin-type lipopeptides from *Bacillus subtilis* QST713. Biochem Biophys Acta 2011;1808: 2000–2008. doi: 10.1016/j.bbamem.2011.04.008 21545788 10.1016/j.bbamem.2011.04.008

[23] Huang CC, Ano T, Shoda M. Nucleotide sequence and characteristics of the gene, *lpa-14*, responsible for biosynthesis of the lipopeptide antibiotics iturin A and surfactin from *Bacillus subtilis* RB14. J Ferment Bioeng. 1993; 76: 445–450.

[24] Yao S, Gao X, Fuchsbauer N, Hillen W, Vater J, Wang J. Cloning, sequencing, and characterization of the genetic region relevant to biosynthesis of the lipopeptides iturin A and surfactin in *Bacillus subtilis*. Curr Microbiol. 2003; 47: 272–277. 14629006 10.1007/s00284-002-4008-y

[25] Blom J, Rueckert C, Niu B, Wang Q, Boriss R. The complete genome of *Bacillus amyloliquefaciens* subsp. *plantarum* CAU B946 contains a gene cluster for nonribosomal synthesis of iturin A. J Bacteriol. 2012; 194: 1845–1846. doi: 10.1128/JB.06762-11 22408246 10.1128/JB.06762-11 PMC3302471

[26] Kunst F, Ogasawara N, Moszer I, Albertini AM, Alloni G, Azevedo V, et al The complete genome sequence of the Gram-positive bacterium *Bacillus subtilis*. Nature 1997; 390: 249–256. doi: 10.1038/36786 9384377 10.1038/36786

[27] Kim SK, Lee NK, Jeong TS, Kim YK, Choi JJ, Bok SH. Structure determination of antifungal KRF-001 produced by *Bacillus subtilis* subsp. *krictiensis*. Kor J Appl Microbiol Biotechnol. 1991; 19: 598–603.

[28] Bok SH, Kim SU, Son KH, Kim SK, Kim YK, Lee HW, et al. Culture of Bacillus subtilis. US patent 5,155,041. 1992.

[29] Kim SU, Lee JW, Lee SH, Bok SH. Identification of bacteria having antifungal activity isolated from soils and its biological activity. Kor J Appl Microbiol Biotechnol. 1991; 19: 337–342.

[30] Cutting SM, Vander Horn PB. Genetic analysis In: Harwood CR, Cutting SM, editors. Molecular Biological Methods for *Bacillus*. John Wiley & Sons; 1990 pp. 65–548

[31] Mead DA, Szczsna-Skorupa E, Kemper B. Single-stranded DNA blue T7 promoter plasmids: a versatile tandem promoter system for cloning and protein engineering. Protein Eng. 1986; 1: 67–74. 3507689 10.1093/protein/1.1.67

[32] Staskawicz B, Dahlbeck D, Keen N, Napoli C. Molecular characterization of cloned avirulence genes from race 0 and race 1 of *Pseudomonas syringae* pv. *glycinea*. J Bacteriol. 1987; 169: 5789–5794. 2824447 10.1128/jb.169.12.5789-5794.1987 PMC214142

[33] Steinmetz M, Richter R. Easy cloning of mini-Tn10 insertions from the *Bacillus subtilis* chromosome. J Bacteriol. 1994; 176: 1761–1763. 8132472 10.1128/jb.176.6.1761-1763.1994 PMC205265

[34] Sambrook J, Fritsch EF, Maniatis T. Molecular Cloning: A Laboratory Manual, 2nd ed Cold Spring Harbor: Cold Spring Harbor Laboratory Press; 1989 pp. 9.31–9.57.

[35] Finn RK. Theory of agar diffusion methods for bioassay. Anal Chem. 1959; 31: 975–977.

[36] Grover M, Nain L, Singh SB, Saxena AK. Molecular and biochemical approaches for characterization of antifungal trait of a potent biocontrol agent *Bacillus subtilis* RP24. Curr Microbiol. 2010; 60: 99–106. doi: 10.1007/s00284-009-9508-6 19777301 10.1007/s00284-009-9508-6

[37] Dunlap CA, Kim SJ, Kwon SW, Rooney AP. Phylogenomic analysis shows that *Bacillus amyloliquifaciens* subsp. plantarum is a later heterotypic synonym of *Bacillus methylotrophicus*. IntJ Syst Evol Microbiol. 2015; 65: 2104–2109. 25835027 10.1099/ijs.0.000226

[38] Yasueda H, Kawahara Y, Sugimoto S. *Bacillus subtilis yckG* and *yckF* encode two key enzymes of the ribulose monophosphate pathway used by methylotrophs, and *yckH* is required for their expression. J Bacteriol. 1999; 181: 7154–7160. 10572115 10.1128/jb.181.23.7154-7160.1999 PMC103674

[39] Earl AM, Eppinger M, Fricke Wf, Rosovitz MJ, Rasko DA, Daugherty S, et al Whole-genome sequence of *Bacillus subtilis* and close relatives. J Bacteriol. 2012; 194: 2378–2379. doi: 10.1128/JB.05675-11 22493193 10.1128/JB.05675-11 PMC3347079

[40] Besson F, Michel G. Isolation and characterization of new iturins: iturin D and iturin E. J Antibiot. 1987; 40: 437–442. 3583913 10.7164/antibiotics.40.437

[41] Besson F, Peypoux F, Michel G, Delcambe L. Identification of antibiotics of iturin group in various strains of *Bacillus subtilis*. J Antibiot. 1978; 31: 284–288. 96084 10.7164/antibiotics.31.284

[42] Tsuge K, Inoue S, Ano T, Itaya M, Shoda M. Horizontal transfer of iturin A operon, *itu*, to *Bacillus subtilis* 168 and conversion into an iturin A producer. Antimicrob Agents Chemother. 2005; 49: 4641–4648. doi: 10.1128/AAC.49.11.4641-4648.2005 16251307 10.1128/AAC.49.11.4641-4648.2005 PMC1280175

[43] Huang CC, Ano T, Shoda M. Nucleotide sequence and characteristics of the gene, *lpa*-*14*, for biosynthesis of the lipopeptide antibiotics iturin A and surfactin from *Bacillus subtilis* RB14. J Ferm Bioeng. 1993; 76: 445–450.

[44] Chen XH, Koumoutsi A, Scholz R, Eisenreich A, Schneider K, Heinemeyer I, et al Comparative analysis of the complete genome sequence of the plant growth-promoting bacterium *Bacillus amyloliquefaciens* FZB42. Nature Biotechnol. 2007; 25: 1007–1014. 17704766 10.1038/nbt1325

[45] Wang X, Luo C, Liu Y, Nie Y, Liu Y, Zhang R, Chen Z. Three non-aspartate amino acid mutations in the ComA response regulator receiver motif severely decrease surfactin production, competence development, and spore formation in *Bacillus subtilis*. J Microbiol Biotechnol. 2010; 20: 301–310. 20208433

[46] Quadri LE, Weinreb PH, Lei M, Nakano MM, Zuber P, Walsh CT. Characterization of Sfp, a *Bacillus subtilis* phosphopantetheinyl transferase for peptidyl carrier protein domains in peptide synthetases. Biochemistry 1998; 37: 1585–1595. doi: 10.1021/bi9719861 9484229 10.1021/bi9719861

[47] Hayashi K, Ohsawa T, Kobayashi K, Ogasawara N, Ogura M. The H_2_O_2_ stress-responsive regulator PerR positively regulates *srfA* expression in *Bacillus subtilis*. J Bacteriol. 2005; 187: 6659–6667. doi: 10.1128/JB.187.19.6659-6667.2005 16166527 10.1128/JB.187.19.6659-6667.2005 PMC1251593

[48] Yang Y, Wu HJ, Lin L, Zhu QQ, Borriss R, Gao XW. A plasmid-born Rap-Phr system regulates surfactin production, sporulation and genetic competence in the heterologous host, *Bacillus subtilis* OKB105. Appl Microbiol Biotechnol. 2015; 99: 7241–7252. doi: 10.1007/s00253-015-6604-3 25921807 10.1007/s00253-015-6604-3

[49] Zhang Z, Ding ZT, Zhong J, Zhou JY, Shu D, Luo D, Yang J, Tan H. Improvement of iturin A production in *Bacillus subtilis* ZK0 by overexpression of the *comA* and *sigA* genes. Lett Appl Microbiol. 2017; 64: 452–458. doi: 10.1111/lam.12739 28374547 10.1111/lam.12739

[50] Koumoutsi A, Chen XH, Vater J, Borriss R. DegU and YczE positively regulate the synthesis of bacillomycin D by *Bacillus amyloliquefaciens* strain FZB42. Appl Environ Microbiol. 2007; 73: 6953–6964. doi: 10.1128/AEM.00565-07 17827323 10.1128/AEM.00565-07 PMC2074971

