# Supplementary figures and images for: Organization and characterization of genetic regions in Bacillus subtilis subsp. krictiensis ATCC55079 associated with the biosynthesis of iturin and surfactin compounds
Source: PLoS One. 2017 Dec 21;12(12):e0188179. doi: 10.1371/journal.pone.0188179 (PMC5739386; doi:10.1371/journal.pone.0188179)

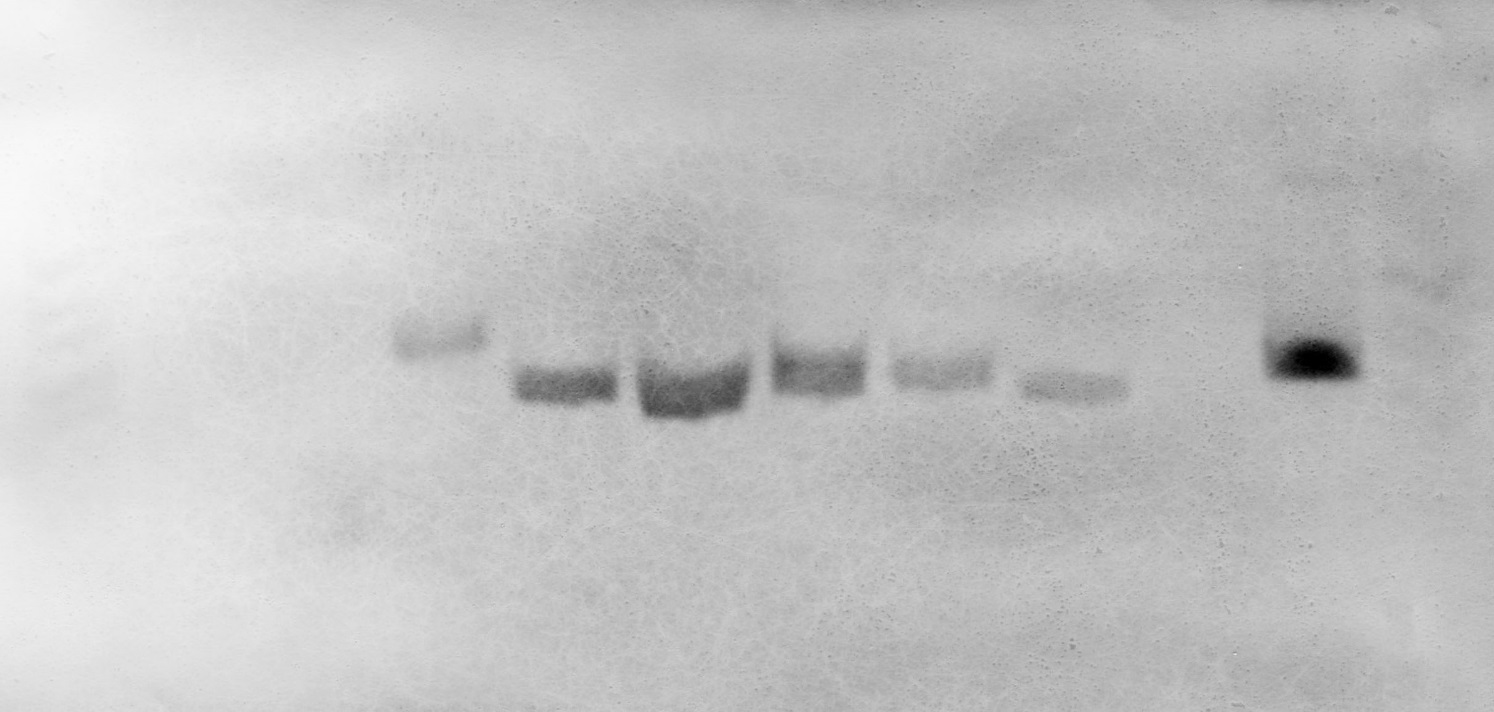


- **1.0kb**

- **1.7kb**


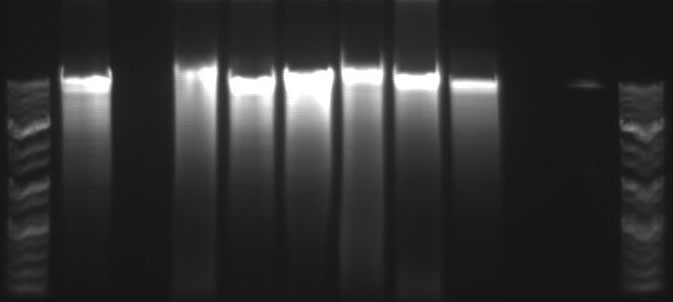


- **1.0kb**

- **1.7kb**

**2**

**3**

**21**

**22**

**23**

**31**

**pJJ121E2-1**

**1**

**S1 Fig**. Young Tae Kim et al.

Supplement: S1 Fig — Lanes: 1, genomic DNA from wild-type B. subtilis subsp. krictiensis digested with ClaI; 2, 3, 21, 22, 23, and 31, genomic DNAs from various transformants digested with ClaI; pJJ121E2-1, the spectinomycin resistance gene from the mini-Tn10 of pIC333 digested with XbaI and BamHI. (DOCX) [file pone.0188179.s001.docx]

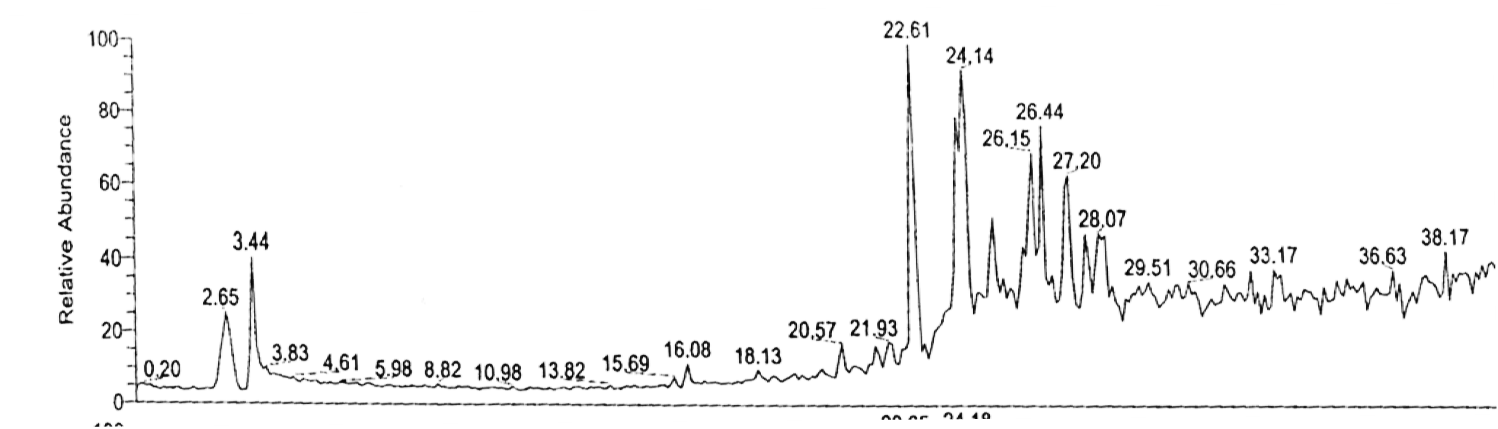


**S2 Fig.** Young Tae Kim et al.

Supplement: S2 Fig — (DOCX) [file pone.0188179.s002.docx]

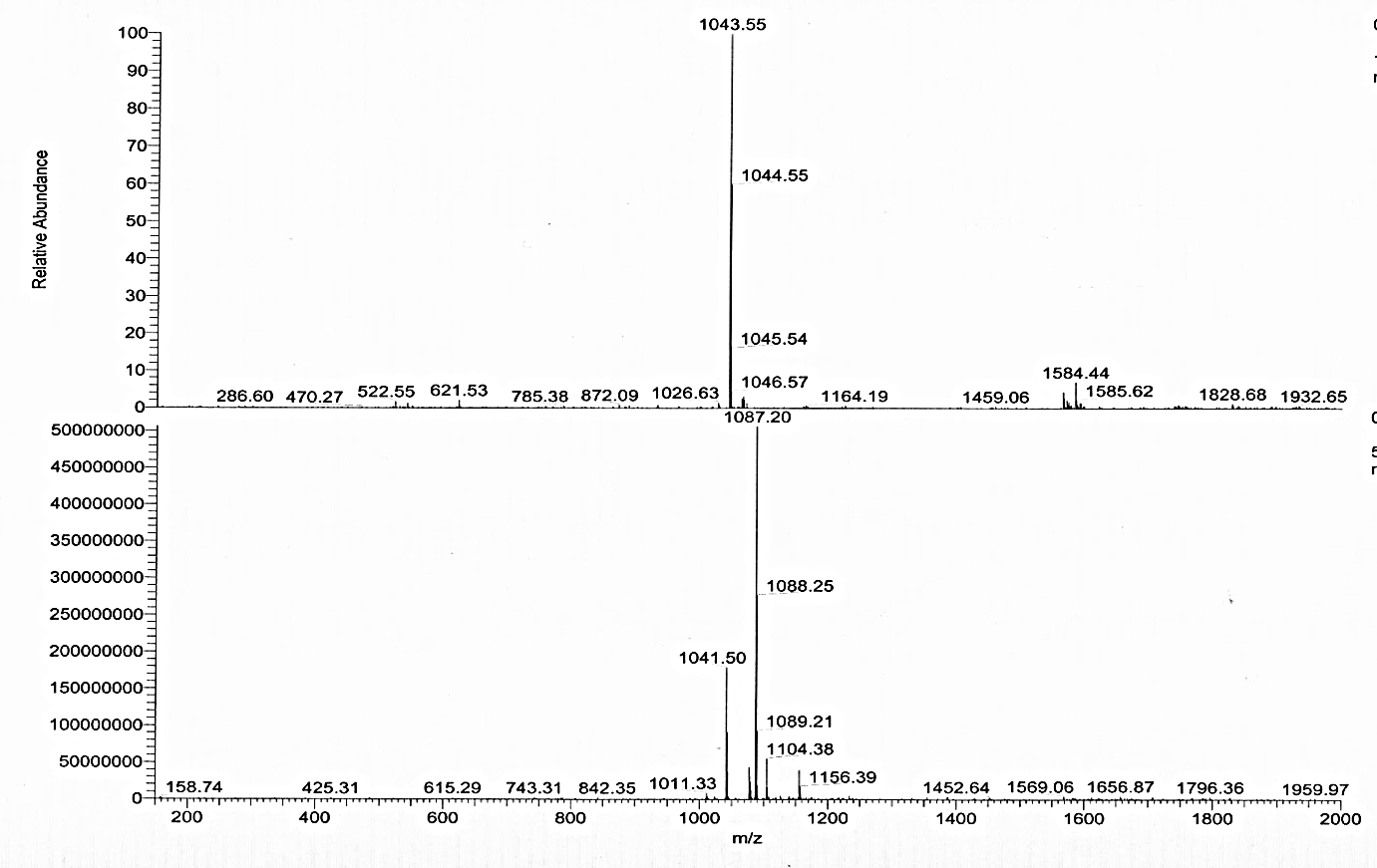


[M-H]-

[M+H]+

**S3 Fig.** Young Tae Kim et al.

Supplement: S3 Fig — (DOCX) [file pone.0188179.s003.docx]

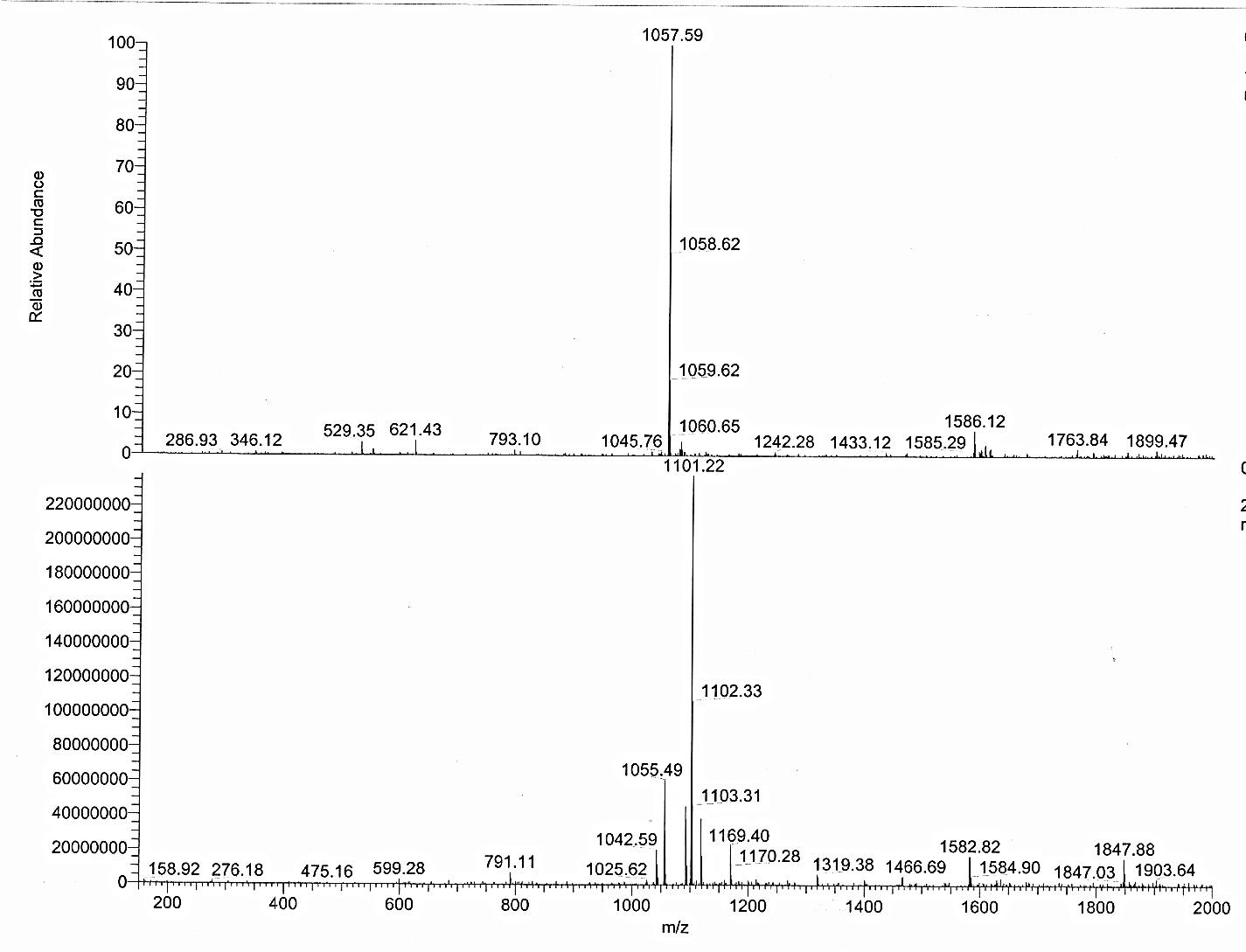


[M+H]+

[M-H]-

[M+H]+

[M-H]-

[M+H]+

[M-H]-

[M+H]+

[M-H]-

[M+H]+

[M-H]-

**S4 Fig.** Young Tae Kim et al.

Supplement: S4 Fig — (DOCX) [file pone.0188179.s004.docx]

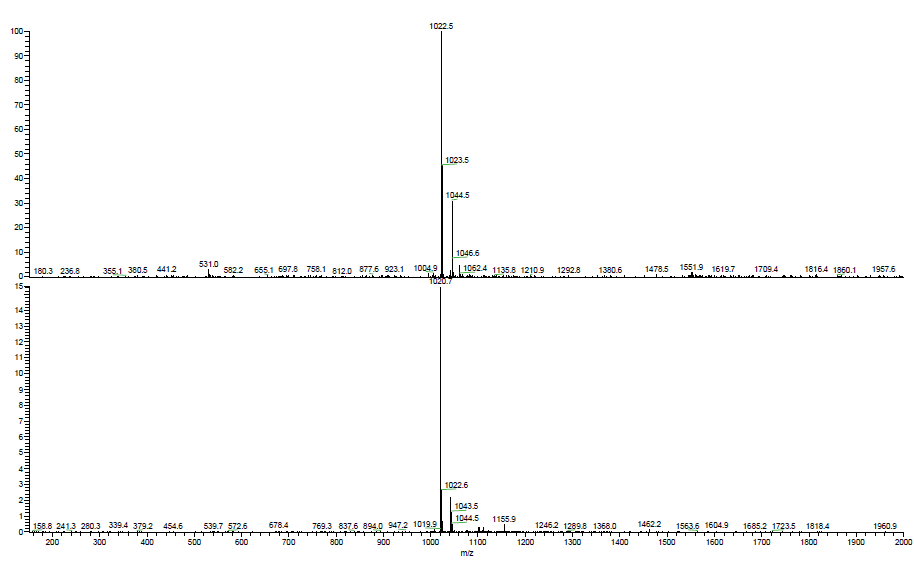


[M+H]+

[M-H]-

**S11 Fig.** Young Tae Kim et al.

Supplement: S11 Fig — (DOCX) [file pone.0188179.s011.docx]

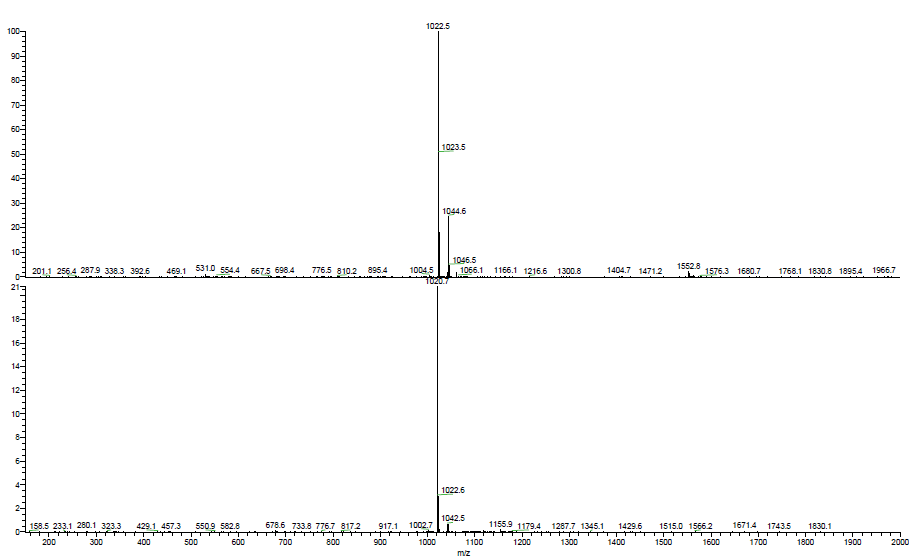


[M+H]+

[M-H]-

**S12 Fig.** Young Tae Kim et al.

Supplement: S12 Fig — (DOCX) [file pone.0188179.s012.docx]

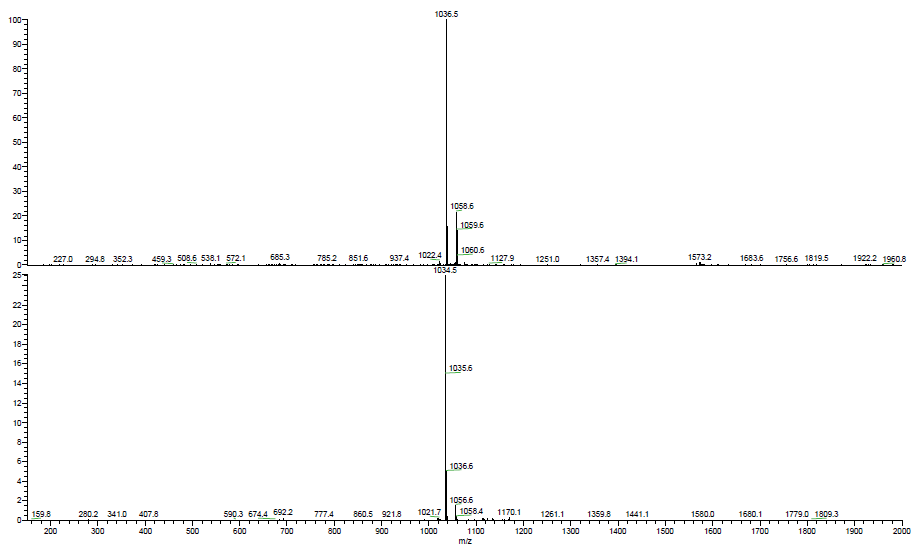


[M+H]+

[M-H]-

**S13 Fig.** Young Tae Kim et al.

Supplement: S13 Fig — (DOCX) [file pone.0188179.s013.docx]
