# Supplementary material for: Organization and characterization of genetic regions in Bacillus subtilis subsp. krictiensis ATCC55079 associated with the biosynthesis of iturin and surfactin compounds
Source: PLoS One. 2017 Dec 21;12(12):e0188179. doi: 10.1371/journal.pone.0188179 (PMC5739386; doi:10.1371/journal.pone.0188179)
Supplement: S8 Fig — (DOCX) [file pone.0188179.s008.docx]

**S8 Fig.** Young Tae Kim et al.
